# A shared future: chemistry's engagement is essential for resilience of people and planet

**DOI:** 10.1098/rsos.212004

**Published:** 2022-04-20

**Authors:** Goverdhan Mehta, Sarah E. Cornell, Alain Krief, Henning Hopf, Stephen A. Matlin

**Affiliations:** ^1^ School of Chemistry, University of Hyderabad, Gachibowli, Hyderabad, Telangana 500046, India; ^2^ Stockholm Resilience Centre, Stockholm University, Kräftriket 2B, 10691 Stockholm, Sweden; ^3^ International Organization for Chemical Sciences in Development, 61 rue de Bruxelles, 5000 Namur, Belgium; ^4^ Department of Chemistry, University of Namur, 61 rue de Bruxelles, 5000 Namur, Belgium; ^5^ Institute of Organic Chemistry, Technical University of Braunschweig, Hagenring 30, 38106 Braunschweig, Germany; ^6^ Institute of Global Health Innovation, Imperial College London, South Kensington, London SW7 2AZ, UK

**Keywords:** chemistry, adaptive capacity, human security, sustainability, vulnerability

## Abstract

Strengthening resilience—elasticity or adaptive capacity—is essential in responding to the wide range of natural hazards and anthropogenic changes humanity faces. Chemistry's roles in resilience are explored for the first time, with its technical capacities set in the wider contexts of cross-disciplinary working and the intersecting worlds of science, society and policy. The roles are framed by chemistry's contributions to the sustainability of people and planet, examined via the human security framework's four material aspects of food, health, economic and environmental security. As the science of transformation of matter, chemistry is deeply involved in these material aspects and in their interfacing with human security's three societal and governance aspects of personal, community and political security. Ultimately, strengthening resilience requires making choices about the present use of resources as a hedge against future hazards and adverse events, with these choices being co-determined by technical capacities and social and political will. It is argued that, to intensify its contributions to resilience, chemistry needs to take action along at least three major lines: (i) taking an integrative approach to the field of ‘chemistry and resilience’; (ii) rethinking how the chemical industry operates; and (iii) engaging more with society and policy-makers.

## 1. Introduction

*πάντα ῥει̃*: Everything flows

          Attributed to Heraclitus, *ca* 500 BC [1]

In a world in which, to an increasing extent, ‘change is the only permanence, and uncertainty the only certainty’ [2], it is not surprising that increasing attention is being given to strengthening resilience in many aspects of life. The need for resilience as a crucial characteristic of people, institutions and systems and as a counterpart to vulnerability [3] has become compelling in the face of many emergent global challenges, including climate change, pandemic outbreaks, looming food and water shortages, fears of reaching environmental tipping points and threats to economic, political and social stability, in addition to the risks posed by many natural hazards. This crucial characteristic needs to be understood as a human purpose requiring decisions by people.

As the science of transformation of matter, chemistry is necessarily located at the heart of responses to many of these challenges, as an essential—but on its own, not sufficient—contributor. Chemistry is able to provide knowledge and materials that can delay, reduce or avoid the build-up of many threats, as well as knowledge and materials that can help reduce and compensate for impacts of unknown or unpredictable shocks that require fast responses. There has been some coverage of roles that chemistry can play in helping build resilience in a few specific and specialized areas [4,5]. Surprisingly, however, hitherto there has been no broad-based discussion of the landscape of ‘chemistry and resilience’.

In this Perspective, we seek to initiate such a discussion by considering the question: *how does and how can chemistry contribute to the resilience of the world in the twenty-first century?* At the outset, we note that, while the response has a technical component that is distinctive to the discipline of chemistry, it must be set in the wider contexts of cross-disciplinary working and the intersecting worlds of science, society and policy, with implications for actors in all these domains.

## 2. The scope of resilience

**Figure 1.**
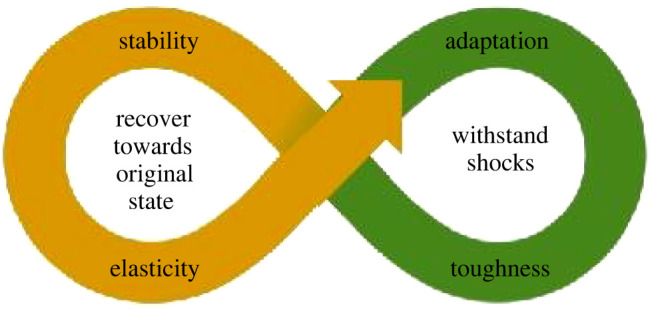
Elasticity and toughness are complementary characteristics of resilience, enabling systems to either recover from a stress or adapt in order to survive.

Broadly, two kinds of complementary definitions of the term ‘resilience’ are used in a variety of everyday and technical contexts. One relates to objects, with resilience being a measurable property that describes the ability to spring back into shape, in some settings corresponding to the property of ‘elasticity’. The other relates to people, their institutions and systems, with resilience being understood as ‘the capacity to recover quickly from difficulties' or, colloquially, ‘toughness’ (figure 1).

Such complementary understandings are seen in the concept of resilience in ecological system responses to natural or anthropogenic changes in ecosystem variables [6,7]. ‘Engineering resilience’ refers to the time required for an ecosystem to return to an equilibrium or steady-state following a perturbation (also defined as ‘stability’). ‘Ecological resilience’ refers to the system's capacity to absorb disturbance and reorganize while undergoing change so as to still retain key properties of function, structure, identity and feedbacks. The latter type links to ‘adaptive capacity’—a capacity to change by blending toughness and elasticity.

In chemistry, the term resilience has been used in a specific technical context to refer to the ability of a material to absorb energy when it is deformed elastically, and release that energy upon unloading. More broadly, however, the underlying concept of resilience as a spectrum from elasticity to toughness has a clear correlation with fundamental notions of equilibrium and transformation. Equilibrium involves perturbed systems' return to a steady-state position following Le Chatelier's Principle, e.g. in the Haber synthesis of ammonia or in the functioning of buffer solutions. Transformation relates to the chemist's use of directed perturbations to disturb equilibrium or to drive a system over a transition state barrier leading to a new reaction product or a changed physical state. Parallels can be seen between these understandings of resilience in chemical phenomena and the forms of resilience attributed to biological systems, where the spectrum extends from homeostasis, as the conserved steady-state position, to change processes including development of the organism and evolution of the species as two essential attributes of life.

Further important conceptual linkages include clarification of the complementarities between resilience, vulnerability and sustainability [3,8,9], necessitating a better alignment between approaches of natural and social/political sciences [3], and acknowledgement that resilience is also a political project [10–12]. It is also vital to recognize the co-evolutionary nature of social and ecological processes related to resilience and development [13].

Overall, a useful composite description [14] of resilience is ***the capacity to withstand shock without permanent deformation or rupture, or to tend to recover from or adjust easily to misfortune or change***. This captures the idea that resilience is not necessarily resistance to undergoing short-term change when a traumatic event occurs, but is rather a flexibility which begins with a deformation and may result EITHER in an eventual move back towards the original condition (elasticity) OR an adjustment, adaptation or evolutionary response to new circumstances (toughness).

The temporal dimension is significant in considering both the building of resilience and the speed with which resilience mechanisms can act when a major deformation occurs. Natural hazards may trigger disasters [15] with little or no warning, infectious diseases can move from a local outbreak to an international health emergency in a few days or weeks, while some economic and environmental changes may accumulate incrementally over years to decades before resulting in severe impacts or reaching tipping points for major disruptions. Each timescale, as well as the degree of predictability of the nature and location of events, requires a different kind of resilience mechanism.

Ways to build resilience involve understanding the structure and behaviour of the system concerned, especially regarding the *connectivity* among parts of the system; its *diversity*, which confers more ways to respond to challenges; and the degree of *redundancy* or available capacity within the system that can be deployed when challenges arise [8]. Resilience building may thus include establishing systems for monitoring and early warning/alert, disaster preparedness and rapid response, risk mitigation and adaptation, as well as stockpiling of materials that are specifically needed at the affected location, such as emergency food, clean water, shelter and medical supplies, or of critical resources of many kinds whose supply chains may be interrupted by large-scale events. Consideration must also be given to the types of skilled human resources, including in different aspects of science and technology (S&T), that are vulnerable to events or that are necessary at different stages in prevention, response and recovery. In the case of outbreaks of new diseases, as the global COVID-19 pandemic has demonstrated, resilience requires having already built mechanisms to ensure that diagnosis, prevention and treatment options can be very quickly developed, produced and distributed on a very large scale. A resilience approach has implications for ensuring reserves, providing training (including simulation exercises), and designing mobilization and protection factors. In all areas, the understanding of temporal dimensions requires coupling with local-to-global systems of planning to ensure that resilience capacities are built, organized and effectively used as needed.

## 3. Framing chemistry and resilience for human security and sustainability

Awareness of vulnerabilities, at scales ranging from personal to planetary, has been heightened in recent years by phenomena that have caused major disruptions to people's lives. Phenomena with dramatically sudden onset and global extent of immediate impact such as economic crises, extreme climate events and pandemics expose both vulnerabilities to shocks and the capacity to cope with them. These recent experiences can serve as experiments that stress-test the resilience of people, institutions and systems [16–19].

A useful framework in which to consider chemistry and resilience in the context of local-to-global vulnerabilities is that of human security, a concept which emerged from the 1994 United Nations (UN) Human Development Report [20] and later UN processes to be defined as ‘freedom from want and fear and freedom to live in dignity’. Its scope includes seven main dimensions that are mutually interdependent: health security, food security, environmental security, economic security, personal security, community security and political security. It accommodates the UN Sustainable Development Goals (SDGs) [21], the Planetary Boundaries framework [22] and the ‘one health’ principle [23] which affirms the fundamental interconnectedness among the health of people, animals and the environment. We have recently presented [24] a description of chemistry's role within the human security framework, highlighting chemistry's central contributions to core aspects including security in health, food, environmental and economic dimensions. This description briefly notes that chemists, as practitioners of the science of transformation of matter, must be guided by the principles of human security while applying their knowledge and skill to developing solutions to the oncoming global challenges and building resilience to changes that are already inevitable. It also emphasizes that human security aims to maximize the stability of the physical systems and diversity of the ecological systems of the planet, aiming towards sustainability as the best practical guarantee of resilience for all.

Chemistry has made large contributions to achieving present standards of human wellbeing and also to providing present levels of resilience to events that may impact on vulnerabilities at scales from personal to global. However—as forcefully demonstrated by contemporary stresses—substantial vulnerabilities remain, threatening sustainability and human security. Chemistry can do much more to increase resilience in many critical areas.

**Figure 2.**
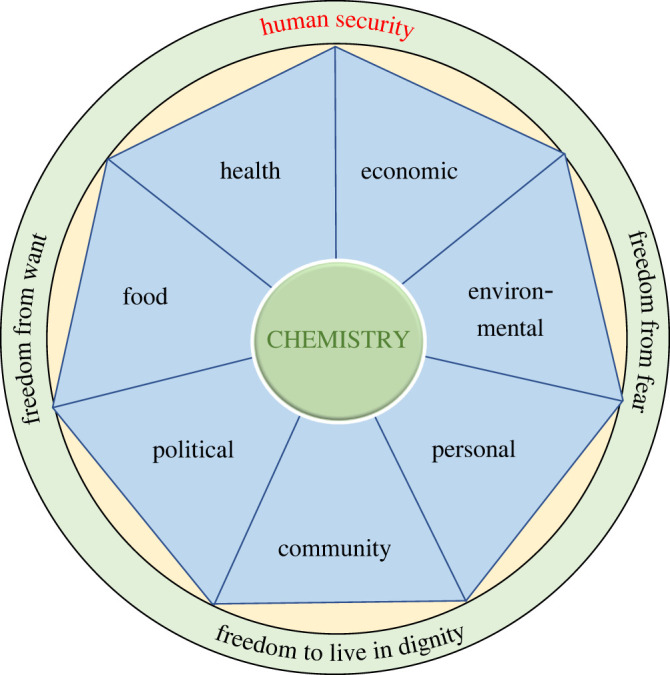
As the science of material transformation, chemistry makes essential contributions to the four material aspects of human security and their interfacing with the three societal and governance aspects.

Resilience can be explored through considering the interdependencies among the dimensions of human security. The interplay of the material dimensions (environment, health, food and economic security) affects the vulnerability and adaptive capacity at all levels from individual people to the whole planet, spanning the linked societal/governance dimensions of personal, community and global political security (figure 2).

As noted by Tanner *et al*. [25], there has been a ‘resilience revolution’ within the sustainable development agenda, fuelling a global journey towards defining, measuring and using the concept of resilience. The goal of achieving resilience is not only about securing freedom from want and fear and freedom to live in dignity in the near term, but also about striving to ensure the means to sustain them in the face of future challenges. Temporally, resilience extends the perspective beyond the SDGs' time-bound staging points, which are mostly set for 2030 as milestones along the route to sustainable development. It offers principles to guide what happens when a new crisis emerges that is not covered by the specific SDG goals and targets; and to guide the choice of new domains to be addressed, new stages of goals and targets to be negotiated as the world sets its horizons beyond 2030.

**Figure 3.**
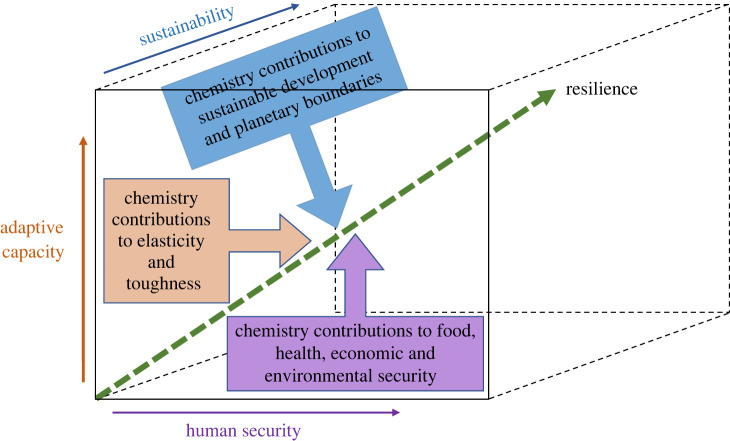
Chemistry contributions to greater resilience as an outcome of strengthening adaptive capacity, human security and the sustainability of people and planet.

In this Perspective, we explore the role of chemistry in relation to strengthening resilience within a framing that takes account of adaptive capacity to counter vulnerabilities across geographical scales and timeframes, aiming at the goal of shared sustainability for people and planet (figure 3).

## 4. How does/can chemistry contribute to resilience?

### 4.1. Resilient and sustainable food systems

Demand for food and better nutrition is rising as the world population grows, incomes rise and dietary and calorie intake patterns change. These trends, along with increasing education, urbanization and globalization, are placing increasing stress on an already stretched global food system. The large increases in production needed to meet world food and nutrition requirements [26] have major economic and environmental implications for expending land areas and water use devoted to agriculture and for increased use of fertilizers and crop protection agents, raising risks to sustainability and resilience. Threats to human security include a very wide range of individual, local and global factors that influence the availability, affordability and nutritional value of food. The interlinkages and cross-dependencies among these factors add great complexity to efforts to achieve resilience on both short- and long-term timescales. Many of these linkages are mediated through chemical processes and products. Important aspects of chemistry's contributions to food science have included provision of knowledge about the molecular structures and nutritional, physiological and metabolic roles of food components, as well as major inputs to expanding agriculture and improving food preservation.

Historically, agricultural production was boosted greatly by the invention in 1910 of the Haber–Bosch industrial process for reacting nitrogen and hydrogen to make ammonia used in fertilizers. Along with improved plant varieties, this process has been credited [27] with providing the food for 40% of the world's greatly expanded population at the end of the twentieth century. However, the process has a high dependence on hydrocarbons (both for production of energy to drive the reaction and as a source of hydrogen for the reaction) and currently accounts for 1.2% of global anthropogenic CO_2_ emissions [28]. Chemistry research has sought ‘green’ options that do not generate CO_2_, for both ammonia and hydrogen production [29–31], as well as methods by which CO_2_ generated can be captured, either for long-term lock-in (e.g. as stable carbonates) or for use in other chemical and physical applications [32,33].

However, even if these carbon emission reduction efforts are fully successful, the large-scale uses of nitrogen fertilizers such as ammonium nitrate and urea contribute many other environmental problems, including eutrophication of waterways, atmospheric pollution, global warming and ozone depletion. Total biogeochemical flows of nitrogen already greatly exceed the Planetary Boundaries safe operating space [22]. Addressing this will require innovative chemistry and materials science to develop less wasteful products and processes for delivering nitrogen compounds into plants and reducing waste and environmental pollution. Similar problems can be seen in the large-scale use of phosphorus-based fertilizers, with the biogeochemical flows of phosphorus also substantially exceeding the Planetary Boundaries safe operating space [22].

In another aspect of agricultural production, application of herbicides and pesticides as crop protection agents provides short-term gains in yield and quality, but can cause long-term damage to ecosystems. Strengthening environmental resilience and sustainability requires the development of more selective products and of new processes for their delivery more precisely to the targeted plants.

Despite efforts at food preservation, the UN Food and Agriculture Organization (FAO) estimates that over 30% of the total supply of food that the world grows goes to waste without being consumed [34]. As well as the implications of this wastage for food security, the decomposition of food sent to landfill or incinerated for energy production is a major source of greenhouse gases which, mostly not captured at present, contribute on a large scale to global warming. Preservation of the quantity and quality of food, from field to fork, has benefitted greatly from chemistry inputs, enhancing food security and resilience to shocks. Notable examples are the development of refrigerants, packaging, inert atmospheres, stabilizers and preservatives to maintain freshness and cleanliness and control decay. However, unintended and undesirable consequences have included the environmental impacts flowing from atmospheric release of refrigerants, such as CFCs, CO_2_ (in ‘dry ice’) and NH_3_, as well as from the environmental release of plastic materials such as packaging and carriers.

A striking example of system interlinkages and cross-dependencies that impact on resilience and supply security has recently been seen in effects of the sudden steep rise in the price of natural gas on the world market. Consequences included threats to the viability of manufacturers of nitrogen fertilizers, because of the strong sensitivity of the economics of ammonia production to the cost of methane [35], which in turn threatened local supplies of CO_2_ being capturing as by-product to be further used in a range of medical and food applications [36]. From the perspective of chemistry and resilience, some important lessons are evident. First, the eventual ‘greening’ of ammonia production will necessitate developing efficient, clean processes to derive CO_2_ from other sources to support a range of important applications such as in health and food. Another lesson is the danger of creating path dependencies or lock-ins [37] when following the precepts of the circular economy, which encourages capture of secondary products from processes and their reuse in high-value applications [38]. While this increases both the economic and atom efficiency of the primary process, it can have the effect of stabilizing existing processes that are potentially harmful both to sustainability and to diverse aspects of human security and serve to weaken resilience to adverse events.

With a growing world population, radical solutions need to be considered to meet the challenge of increasing the security and resilience of food. The goal must be expanding production of food that contributes to a healthy diet [39] while decoupling it from other critical resources such as land and water, from excessive levels of biogeochemical flows of nutrients such as nitrogen and phosphorus compounds and from environmentally damaging outputs to land, aquatic and atmospheric systems. Solutions may come, among other strategies, from diversification of foods [40], immunity-boosting foods [41], food loss and waste reduction and recycling [42,43], growing crops resistant to drought, climate change and pests [44–46] including using transgenic plants [47], dense foods [48] and preservation additives for prolongation of shelf life without recourse to dry ice and refrigeration [49,50]. Conventional agriculture approaches may need to superseded, at least in part, by alternatives that are both efficient and ensure targeted inputs and controlled outputs. Pointers to such approaches, where chemistry makes important contributions, include the burgeoning field of hydroponics to produce plants. This requires efficient recycling of hydroponic waste solution chemicals to ensure environmental and ecosystem protection and conservation of mineral resources [51], and there is also potential for the synthesis of key feedstocks, such as the preparation of amino acids from CO_2_ [52]. An alternative for the production of meat is the use of scaffolded systems for culturing of meat cells, with chemistry's numerous contributions including microcarrier materials [53], while advances are also being made in the culturing of cell-based seafoods [54]. Chemistry provides essential tools in ensuring the quality and lack of toxicity of foodstuffs [55] as well as synthetic food flavours and colourings.

It has been argued [24] that a comprehensive new approach should be developed to ‘chemistry and food’ that integrates food chemistry more closely with mainstream chemistry. The aim should be to provide chemists with the breadth of knowledge and research training necessary to make front-line contributions on food, health and environment that will help to solve resilience challenges.

### 4.2. Telecoupled water systems

Water intake is essential for human life and water is key to food production. Yet, while the 1948 Universal Declaration of Human Rights affirmed the right of everyone to adequate food, it was not until 2010 that the UN General Assembly declared access to clean drinking water and sanitation as a human right [56]. Consequently, water security [57] is often discussed as a component of food security [58]. Agriculture requires large quantities of water for crop irrigation and also clean water for livestock and various production processes. Irrigation consumes about 70% of all freshwater appropriated for human use, including in the production of food and non-food crops such as cotton, rubber and industrial oils for heavy-duty lubricant applications.

The high dependence of many systems on water [59,60] places this substance at the centre of concern within human security and environmental sustainability [61]. Intricate and complex interconnections among water-dependent systems—including those involved with vegetation and soil, run-off, transpiration and atmospheric rivers—have implications within and far beyond localities and contribute to planet-wide phenomena. For example, long-distance processes called telecouplings significantly influence the state of local groundwater bodies, with implications for water security [62].

With global water demand increasing at about 1% per year over recent decades and expected to continue growing significantly for the foreseeable future [63], demand for water is projected to exceed supply by 40% in 2030 if no changes are made in how water is managed [64]. Resilience of security in water supplies is intimately connected to a range of factors including expanding demand for domestic, agricultural and industrial uses, increasing urbanization and the impacts of climate change, environmental pollution and geopolitics [11,65].

Strengthening resilience in the face of the oncoming challenges will require intensified efforts to ensure water quality and to innovate large-scale and environmentally benign processes for the desalination of seawater [66] and purification of wastewaters [67], as well as for the development of cleaner industrial processes that create less contamination and require less abstraction and consumptive use of water in the first place [68]. Chemistry roles include the identification, quantification and removal of toxic contaminants, such as arsenic, fluoride, a variety of heavy metals and microbes [69], and applications of nanotechnology [70], such as nanofiltration membranes involving graphene [71] and other materials [72], to produce drinking water and supermagnetic and other iron oxide nanoparticles for decontamination of water and for wastewater purification and recycling [73,74]. Processes for harvesting water from atmospheric moisture, including through the use of hydrogels, other polymers and metal-organic frameworks are options being explored [75,76].

### 4.3. Healthy people, healthy planet

Chemistry's contributions to the 2½-fold increase in average global life expectancy in the last two centuries [77] have included the provision of fundamental, molecular-level knowledge concerning the nature of diseases and metabolic process and the practical development of diagnostic, preventive and treatment approaches [78]. An expanding array of novel vaccines [79] to prevent communicable diseases and the availability of a pharmacopoeia of antimicrobial drugs to treat infections have been notable chemistry-based innovations that have enhanced individual health and also increased health security at population levels.

However, major vulnerabilities exist, which chemistry needs to help address. One concerns the range of communicable diseases for which adequate diagnostic, preventive and curative tools are available. For bacterial diseases, the combination of spreading antimicrobial resistance and declining rates of discovery of new antibiotics has led to severe depletion of the list of effective drugs to treat these infections, widely seen as a crisis for twenty-first century medicine [80]. For viral diseases, the equivalent of the broad-spectrum antibiotic is not known [81], and there has been limited success to date in developing curative drugs for individual classes of viral infections. Alarmingly, it has been estimated that 1.7 million currently undiscovered viruses are present in mammal and avian hosts, with up to about half of these potentially having the ability to infect humans [82]. More broadly, approximately 30 000 diseases of all kinds in human beings have been recognized to date, but only about a third of these can be adequately treated at present [83]. Addressing these gaps in the scope of available treatments requires investment in a range of physical and biomedical sciences to expand the S&T options [84] and to build a broad spectrum of human and infrastructural resources, but also to improve the systems of innovation, regulation and reward to ensure that the ecosystem of disease diagnosis, prevention and treatment works for the benefit of people everywhere [85].

Another vulnerability concerns known inadequacies in the global systems that are intended to provide health security in the face of infectious disease threats. The independent global Monitoring Board on global pandemic preparedness cautioned [86] in 2019 that the world was ill-prepared for a pandemic. The appearance of COVID-19 in late 2019 and its elevation to pandemic status within a few months demonstrated the correctness of this assessment, with the 2020 report of the Monitoring Board describing a world in disorder [87].

An important element of the rapid response systems required to provide resilience to pandemics is the capacity to rapidly develop, scale up and manufacture essential public health and medical materials. In the case of the response to COVID-19, chemistry was the immediate source of environmental disinfectants and antiseptic agents for hand cleansing, a contributor to the rapid production of diagnostic tests ranging from home kits based on lateral flow to laboratory analyses utilizing polymerase chain reactions and the source of techniques for molecular-level manipulations and physical chemistry that underpinned very rapid creation of novel vaccine structures and their incorporation into practical delivery vehicles, such as nanoparticles [88].

However, the pandemic has also exposed weaknesses in the systems needed for resilience. One of these is evident from the contrast between the speed with which novel vaccines were developed, licensed, manufactured on a large scale and rolled out to the public, and the relatively slower pace at which novel drugs were brought to clinical use. While the repurposing of existing drugs such as dexamethasone, favipiravir, remdesivir and lopinavir–retinovir has had significant impact on survival for those with severe COVID-19 infection, it was 2 years after COVID-19's emergence that the first oral antiviral drugs (molnupiravir [89] and nirmatrelvir/ritonavir [90]) were licensed for the prevention of severe illness in COVID-19 infection. 4′-Fluorouridine [91] and various antibody cocktails [92] also hold promise for interrupting the course of infection. An accommodative stance by drug regulators in granting emergency-use authorization for repurposed and new drugs is a major change that contributes to resilience in pandemic times. However, there are concerns about availability and affordability of these emerging treatments to a cross-section of populations [93]. Solving the challenge of global access, which sits at the junction of S&T, economics and politics, will be crucial if the world is to ensure health security and resilience to pandemics by acting on the recognition that ‘no-one is safe until everyone is safe’ [94].

As expressed in the ‘one health’ concept, the health and wellbeing of human beings are intimately connected with those of animals and the environment [95]. Examples of the close interlinkages among these three domains include the spread of antimicrobial resistance to antibiotics [80] and the impacts of climate change on human and animal health [96]. It is evident that building resilience in each of these dimensions requires closely coordinated attention to all three and a convergent approach is likely to be most effective and economical. Investment in building a much more broadly based system for developing products to promote, protect and restore health can, if deliberately shaped, provide the basis of diverse knowledge, tools and methodologies that can be very swiftly co-opted to generate rapid responses to new health threats that emerge. This approach requires not only attention to innovation infrastructure but also ensuring an adequate supply of human resources. We have previously argued [97] the need to create a new, defined discipline of ‘chemistry and health’ to equip chemists with the necessary range of knowledge and skills.

A radically reformed system can also address two further vulnerabilities with additional implications for community and political security—vulnerabilities that are seen in the infrastructures that relate to availability of health products. (i) For many years, critics of the existing patent system have argued that, while it has provided a stimulant to innovation to some degree [98], it has not served the health of humanity as well as needed [99] and has especially failed to benefit those affected by ‘diseases of the poor’. Recent decades have seen efforts to develop alternative innovation models to address this challenge [100–102]. The need for rapid access by the poor—and especially those in lower-income countries—to products required for dealing with health emergencies and crises has been brought into sharp focus by the COVID-19 pandemic. Despite international commitments to support the COVAX mechanism [103], and 55% of the world population so far receiving at least one dose of a COVID-19 vaccine, only 6.2% of people in low-income countries had received at least one dose by December 2021 [104]. (ii) Improving the resilience of lower-income countries towards health threats must include building their domestic capacity for the discovery and production of a wide spectrum of health products, coupled with changes that will enable production outsourcing, technology sharing and compulsory licensing. The contribution of chemistry to this resilience strengthening must include education and training of scientists and technicians [105], as well as helping to build scientific literacy in communities as a social capability [106] to help counter misinformation.

### 4.4. Securing sustainable economies

A cross-over point was reached in 2020, when it was estimated [107] that the global mass of human-made material exceeded all living biomass, for the first time in our planet's history. Chemistry's roles in the global economy include providing many materials that are vital to everyday life, as well as employment and value generation. One assessment found that, in 2017, the international chemical industry contributed an estimated US$ 5.7 trillion to global economic output, or 7% of world GDP, through direct, indirect and induced impacts, and supported 120 million jobs worldwide [108], touching nearly every goods-producing sector in the world. Yet, here too there are vulnerabilities. Some are inherent in contemporary economic practice and some are specific to chemistry—but both types have implications for resilience related to human security and sustainability.

The following interconnected factors in contemporary economic practice are especially prominent sources of vulnerability:

(1) *Lean and globally interdependent supply chains.* Efforts that aim at continuous flow processing with low inventory volumes, just-in-time production and accurate scheduling of transport have become popular as a means to achieving cost-effective and responsive supply chains [109]. However, these measures also have the effect of eliminating redundancy in the system with implications for resilience since they reduce the system's buffer capacity to respond quickly to system disruptions. In a 2021 survey of more than 1000 businesses from across 10 industry sectors in the UK and USA, supply-chain vulnerability was classed as one of the most high-risk, low-resilience problems, with 31% ranking this as their top boardroom risk concern, and ‘agility in supply chains' being emphasized as a key response [110]. The worldwide interruptions to employment, production and transport caused by COVID-19 created serious supply shortages in many areas, including foodstuffs, materials for health and microprocessors (with cascading effects on many products that require them, including computers and cars). The accident in which a single container ship blocked the Suez Canal, interrupting around 13% of world trade for several weeks, provided another demonstration of supply-chain vulnerability [111]. Strategies for increasing supply-chain resilience by ensuring diversity of supply sources are receiving increasing attention, including the roles of public policy and international cooperation [112].
(2) *Outsourced production.* The practice of outsourcing some aspects of a manufacturing chain to external parties is seen to have a number of business advantages [113]. However, especially when the sources are offshore, this also creates vulnerabilities, including loss of control, potential for lower quality and risks to supply chains. As well as potentially weakening manufacturing resilience, the decoupling of R&D and other technology aspects from later steps [114] may also lead to loss of innovation capacity in a company or country [115]. Reconnecting these competences helps maintain agility and build resilience when threats require new or adapted products to be developed rapidly in response to an emergency.

The interface between S&T and economic and socio-political policies also creates vulnerabilities that, among other areas, affect industry. One aspect of this concerns the supply of skilled human resources for research (both in academic and industry), innovation and production. Shortages of highly trained scientists and technologists, which may result from policies leading to under-investment in science education as a push-factor or unattractive career compensations as a pull-factor, are offset by policies enabling or encouraging recruitment from overseas of advanced students by universities and of fully trained scientists by industry. This leads to brain-gain on one side (usually in richer countries) and brain-drain (a long-term loss of technical skills, creativity and stimulus for innovation) on the other. The overall shortage of skilled human resource for S&T increases the vulnerability of both sides, weakening their capacities to respond rapidly to situations that require immediate, intensive S&T effort. Resilience requires that every country aims to develop its own domestic supply of skilled human resource for S&T. This must involve widening basic and advanced education in S&T, offering fulfilling career pathways and ensuring equality of access and advancement, at all stages, to the full diversity of the population, which provides for greater capacity to respond to challenges.

The resilience of the chemical industry includes an internal concern in relation to threats to its own survival and profitability, as with any business; an external concern with local to global dimensions, given the central importance of chemical products to every aspect of human activity; and as a corollary of this, concern with chemistry's positioning as a key asset to support the resilience of many other facets of human security and sustainability [116–118].

One important linkage that cross-cuts these dimensions relates to critical chemicals—the recognition that elements are present on the Earth in finite amounts and, in practical terms, have limited availability for exploitation [119]. There is a growing awareness that dependence on elements that are found in low abundance in the Earth's crust are present in appreciable concentrations at only a few locations, and that are being considered for very large-scale applications implies a risk of exhaustion of supply or potential for supply-chain disruptions. Descriptions of such materials as ‘critical’ or ‘endangered’ [120] and the policy interest shown in ‘strategic’ elements [121] represent signals that resilience strategies need to be developed.

— Part of the solution to scarcity must come from recovery and recycling of as much material as possible, with this being built into the design of products and processes from the outset [122], with key roles being played by policies [123] as well as by S&T. Examples of the science solutions being explored include recycling of lithium from batteries [124] and of rare earth metals that are critical to many current electronic devices [125]. However, material recovery itself requires expenditure of more energy and materials, and there is always some material dispersal that makes recovery impractical (material entropy), so that recycling needs to be coupled with, or as far as possible replaced by, minimizing material consumption and waste in the first place [126]. Such considerations are central to understanding chemistry's role in the circular economy and building on this, and the recent emergence of circular chemistry [127], to develop the circular chemical economy approach to transform the chemical industry into a ‘fossil-independent, climate-positive and environmentally friendly circular economy’ [128].
— Concerned about the resilience of supply and the sustainability of critical chemicals in the European Union, the European Commission published a chemicals strategy for sustainability in 2020 [129]. The Strategy recognizes that resilience to supply disruptions is vital in many areas of human wellbeing and sustainability, including technologies for climate neutrality, such as batteries, wind turbines and photovoltaics, as well as for clean material circularity. It calls for ‘diversified sources of supply and a better management of the risk of disruption at all levels, strategic reserves and stockpiling, as well as mechanisms to ensure that supply chains can continue to operate unaffected in case of a crisis.’ It also notes that ‘The transition to chemicals that are safe and sustainable by design is not only a societal urgency but also a great economic opportunity, as well as a key component of EU's recovery from the COVID-19 crisis’.

Another important linkage concerns over-production, whether or not the production is limited by present availability of source materials. The Planetary Boundaries framework [22] has drawn attention to the sustainability problems that are caused when global biogeochemical flows of key materials exceed the safe operating limits of critical Earth systems. One of the Planetary Boundaries concerns the role of Novel Entities and, while these remain to be fully defined, it was recently proposed that plastics be considered as one of the indicators for this category—and it was assessed that plastics already exceed the safe operating limits [130], with impacts on many aspects of atmospheric, aquatic and land-based systems. This example illustrates the complexity of the challenges (including the vast array of applications of plastics; poor control over waste management processes; lock-in to processes that have short-term economic attractiveness and may also cause product dependencies; and resistance to change on the part of industry or consumers). It also illustrates the diverse potential roles for chemistry in devising sustainable, resilient solutions—ranging from production of plastics from renewable bio-sources to design of biodegradable and recyclable materials that avoid combustion or degradation liberating CO_2_.

Analyses suggest that chemical industry companies that did well through the recent period of stresses had learned resilience lessons from previous economic downturns. They were agile in cost-cutting and restructuring proactively, were adept at managing stocks (e.g. managing volumes or production location to match shifting demand) and flows (e.g. changing supply routes to guarantee supply of consumer goods or intermediates), at using new (including digital) technologies and process designs, and working with small and medium size enterprises [131,132] (https://www.weforum.org/focus/chemical-industry-and-societal-resilience). While resilience of individual companies to diverse challenges may be regarded by some as a commercial matter to be left to market forces, it is evident that consequences can flow to a wide range of aspects of human security and sustainability when a pivotal company or sector is weakened or eliminated. In the case of the chemical industry, weaknesses may emerge from low levels of R&D investment and/or a poor environment for innovation—areas in which there needs to be engagement by both industry and the public sector [133].

### 4.5. Building resilience for a sustainable planetary environment

There has been mounting evidence of the extent of environmental damage caused by human activity that began to gather momentum with the industrial revolution and greatly accelerated since the mid-twentieth century—signposted in the proposal [134] to designate the current period as the Anthropocene Epoch, in which human beings have become the dominant source of changes to the planetary environment. Humanity's capacity to transform matter has, unfortunately, not been matched by attention to preventing consequent environmental impacts. Combining traditional categories of hazard, exposure and vulnerability, ‘Anthropocene risk’ has been proposed [135] as a new category, defined as those risks that originate from, or are related to, anthropogenic changes in key functions of the Earth system (such as climate change, biodiversity loss and land-use change). They emerge due to the evolution of globally intertwined social–ecological systems and exhibit complex cross-scale interactions. These range from local to global, and short-term to deep-time (millennia or longer). There is now considerable risk that the Earth's environment will be severely degraded during the twenty-first century, possibly to the stage where a variety of tipping points will be passed that will result in very long-term changes and produce an environment that is much less hospitable to human beings and to many other species [136,137].

Building resilience to today's globalized threats is a collective challenge for all of science, society and politics. In the domain of ensuring environmental security, it must incorporate innovations from chemistry of at least two kinds, as part of a collective effort:

#### 4.5.1. Building capacity to withstand shocks

Chemistry's capacity for material transformation needs be applied in many critical areas that avert, delay or lessen the severity of looming changes. It is vital to take an integrative systems approach in the evaluation of such possibilities, since all of them require the sourcing and transformations of materials and the disposal or recycling of many intermediates, by-products and waste. One promising approach is the concept of Total Material Requirement, which has been used, for example, to assess the total material requirement for the global energy transition to 2050 [138]. Such systemic considerations must also be carefully applied to the question of how to replace fossil-fuel-based plastics, whose massive impacts on the global environment are also demanding urgent attention. Chemistry can offer a range of bio-based plastics, with a particular focus on renewable sources and potential for recovery, recycling and low environmental impact.

Another area with major environmental implications where chemistry can make a difference is in finding alternatives to the production of concrete, which is considered to be the most widely used substance in the world after water [139]. Concrete is traditionally a mixture of an aggregate (coarse- to medium-grained particulate materials like sand, gravel and stone) bonded together with a cement paste that hardens over time. Production of the cement component involves a high energy input to reach the required temperature for the decomposition of calcium carbonate, and currently liberates large amounts of CO_2_, leading to net 8% of global emissions. Meeting the ambitions of the Paris Agreement on climate change will necessitate major reductions in the absolute amounts of these cement-related emissions, while at the same time the global demand for concrete is increasing. Chemistry and engineering solutions being developed include more efficient and environmentally benign processes (e.g. increasing the energy efficiency of cement plants, replacing fossil fuels with alternatives, and capture and storage of CO_2_ emitted) and novel concretes that have intrinsically lower carbon footprints [140,141] or that are cured with injections of CO_2_ [142].

Chemistry is also working to develop approaches to the clean-up of existing levels of environmental contamination, including developing ways to sequester CO_2_ from the atmosphere. This is considered essential if global warming targets are to be met by mid-century [143,144]. This could provide a means of increasing resilience of the planetary environment to the threat of extreme weather events and to the risk of triggering a climatic tipping point during the transition period. It remains to be seen whether such approaches can be scaled to a level that will have significant impact on atmospheric CO_2_ and also whether the material and energy demands of the sequestering systems themselves would be acceptable.

#### 4.5.2. Building capacity to adjust and recover from change

Chemistry can contribute to adaptive mechanisms to improve chances of survival. Much of the increase in consumption of energy and materials witnessed in recent decades has been associated with the increasing urbanization of the world's growing population. Currently about 55% of the world's population lives in urban areas, expected to increase to 68% by 2050 [145]. An expanding movement for ‘resilient cities’ emphasizes building resilience to the growing twenty-first-century physical, social and economic shocks and stresses [146,147]. There has been some coverage of roles that chemistry plays in helping build resilience in a few specific areas, such as the development of high-performance products and technologies by working with architects and designers to make building materials more energy-efficient, waterproof, health-promoting and resilient to natural disasters [4,148,149], developing more heat-resistant materials for a wide range of applications including in travel and transport (e.g. reflective, cooler pavements, melt-resistant asphalts, buckle-resistant rails), roofing (heat-reflective roofs), floodproofing barriers and fireproof building materials [150]. Chemistry has also been involved in demonstration of resilience in recovery from flooding by contributing to the field of conservation science [5]. A more comprehensive effort is needed by chemists, in collaboration with all other relevant sectors, to take stock of the range of city inputs and outflows of materials and energy, with a view to addressing short-, medium- and long-term approaches to strengthening resilience, taken in both a local and planetary context.

## 5. Conclusion and way forward

Use of the term resilience may give rise to policy responses that address external shocks…

by fighting symptoms rather than tackling root causes.

          Krause, UN Research Institute for Social Development [151]

The foregoing discussion highlights an important characteristic of resilience in complex systems that has been recognized in other contexts: resilience is a function that emerges from the working of the whole system and is not simply a property that can be attributed to some part of the system in isolation [152]. It is therefore necessary to take a systems approach and adopt ‘resilience thinking’ in analysing and enhancing resilience [153]. Moreover, the function of resilience may be viewed as a system ability rather than an outcome [154].

Chemistry has many key roles to play in strengthening resilience to threats and vulnerabilities, at scales from local to planetary and at levels from individual to population. Critically important aspects where chemistry's contribution is central include the interplay of food, health, economic and environmental security. Chemistry must not only continue to make innovative inputs within each of these areas, but now needs to substantially raise its game, to intensify its inputs into integrative efforts to building resilience and achieving sustainability.

This will require action along at least three major lines which will take chemistry into less familiar territory:

(1) *Taking an integrative approach to the field of ‘chemistry and resilience’*. This implies much more than creating a list of all the individual materials, processes and applications involved in resilience to threats to human security and sustainability, so they can each be looked at. It requires that chemists: (i) change their own perspectives and processes, beginning with acquiring competence in systems thinking and cross-disciplinary working; (ii) adopt and further develop and extend available tools that chemistry has been shaping (e.g. green, sustainable and circular chemistry) to factor in the impacts of material sources, reactions, processes, products, uses and disposals on human security and sustainability; (iii) further develop priority rankings that go beyond the existing parameters (e.g. the waste hierarchies and ladders based on criteria such as value chains, atom economy, energy efficiency, recapture, reuse and recycling rates) to also now include resilience-related characteristics that in turn need to be defined from a chemistry perspective. These might include risks to supply due to absolute scarcity and regional concentration, sensitivity of process to energy or co-supply factors, danger of lock-in to particular secondary sources or production processes, and the speed and technical ease with which processes and products can be adapted to new circumstances.
(2) *Rethinking how the chemical industry operates*. New ‘business as usual’ models are needed, in which the shifting vulnerabilities of sources, supply chains, economic and workforce factors and the resilience benefits of ensuring agility to deal with risks on short- to long-term timescales are given increased priority in the ever-present competition with near-term profits.
(3) *Engaging with society and policy-makers.* One of the most pervasive and challenging features of resilience is that building resilience is ultimately a political choice. Whether in national or global contexts, including through using S&T, this choice is influenced to varying degrees by societal views. The potential benefits to resilience in human security and sustainability that can be derived from S&T will not be achieved without the active participation of diverse societal actors in multiple political processes, and without taking account of systemic factors that may serve as enablers or barriers to strengthening resilience [155]. The chemistry community needs to greatly expand its engagement in this science-society-policy nexus, learning to communicate in non-technical language to inform, and argue the case for policies, investments and reprioritizations that will favour strengthening resilience.

Finally, it is important to be aware of what resilience cannot do—which is to provide a moral and ethical compass for humanity. With that compass pointing the way, resilience thinking can provide the inspiration for devising the technical means, across all spheres, to achieve the goal. We hope that this Perspective will stimulate readers to reflect deeply on the roles chemists and their discipline can play in contributing a more resilient future for people and planet.

## Data Availability

This article has no additional data.
